# Psychosocial, Environmental, and Functional Capacity Determinants of Psychological Workload in Retail Workers: A Multidomain Assessment Using a Digital Tool

**DOI:** 10.3390/ijerph23060774

**Published:** 2026-06-08

**Authors:** Pongjan Yoopat, Nisakorn Julraksa, Weerawat Liemmanee, Karn Yongsiriwit, Thannob Aribarg

**Affiliations:** 1 Ergonomics Unit, Department of Medical Science, Faculty of Science, Rangsit University, Pathumthani 12000, Thailand; 2 Department of Mathematics and Statistics, Faculty of Science, Rangsit University, Pathumthani 12000, Thailand; nisakorn.j@rsu.ac.th (N.J.); weerawat.l@rsu.ac.th (W.L.); 3 College of Digital Innovation Technology, Rangsit University, Pathumthani 12000, Thailand; karn.y@rsu.ac.th (K.Y.); thannob.a@rsu.ac.th (T.A.)

**Keywords:** occupational stress, psychosocial factors, workplace bullying, environmental stressors, functional capacity, grip strength, digital health, retail workers, public health surveillance

## Abstract

**Highlights:**

**Public health relevance—How does this work relate to a public health issue?**

- Retail workers face multidomain occupational exposures—psychosocial, environmental, and physical—that elevate psychological workload and threaten workforce health at a population scale.
- This study demonstrates that a digital tool (the Find My Stress PWA) can capture these exposures simultaneously in real-world field settings, addressing a critical gap in public health surveillance capacity.

**Public health significance—Why is this work of significance to public health?**

- Six independent predictors of psychological workload were identified—including workplace bullying, postural difficulty, thermal discomfort, air quality concerns, task duration, and grip strength—providing an evidence base for targeted, multidomain occupational health interventions.
- The Find My Stress PWA showed excellent reliability (α = 0.97) and high user acceptance (87%), establishing it as a scalable, low-burden screening platform suitable for large-scale occupational health monitoring programs.

**Public health implications—What are the key implications or messages for practitioners, policy makers and/or researchers in public health?**

- Handgrip strength measurement should be incorporated into routine workplace health monitoring as a simple, low-cost functional indicator that can signal early risk of psychological overload before clinical symptoms emerge.
- Policymakers should integrate multidomain digital stress screening into national occupational health surveillance frameworks—particularly for the retail sector—where psychosocial and environmental health risks are systematically underdetected.

**Abstract:**

Retail service workers face complex occupational demands across psychosocial, environmental, and physical domains; however, integrated multidomain workload assessments remain limited. A cross-sectional study among 253 retail workers used the Find My Stress Progressive Web Application (PWA)—a digital tool assessing subjective workload (Subjective Workload Index; SWI), psychosocial factors, environmental discomfort, musculoskeletal symptoms, and handgrip strength. Hierarchical multiple regression identified four significant SWI predictors: postural difficulty (β = 0.176, *p* = 0.012), workplace bullying (β = 0.175, *p* = 0.008), task duration (β = −0.179, *p* = 0.004), and air quality (β = 0.171, *p* = 0.011; Adjusted R^2^ = 0.199, ΔR^2^ = 0.227, *p* < 0.001; VIF: 1.03–1.57). Grip strength was retained as a functional capacity indicator. Sex-stratified analyses revealed distinct risk profiles: postural difficulty and task duration predicted SWI in men (Adjusted R^2^ = 0.224); workplace bullying was the sole predictor in women (Adjusted R^2^ = 0.170). The PWA demonstrated excellent reliability (α = 0.97) and usability (87%; *n* = 359). The Find My Stress PWA provides a scalable platform for multidomain stress screening. Integrated ergonomic, organisational, and environmental interventions guided by digital screening offer targeted strategies for reducing occupational workload burden in retail settings.

## 1. Introduction

Retail service employees constitute one of the world’s largest and most rapidly expanding occupational groups, yet they remain among the most understudied from a public health perspective [1,2]. Recent evidence confirms that psychosocial risk factors—including excessive workload, interpersonal conflict, and job insecurity—continue to disproportionately affect retail employees, with work stress and technological disruption emerging as compounding occupational hazards in this sector [3]. Customer-facing duties, prolonged standing, manual handling, and high-volume, variable workloads collectively contribute to elevated fatigue and psychological strain [4]. These psychosocial demands frequently co-occur with adverse environmental conditions—including thermal discomfort, poor air quality, noise, and crowding—that independently amplify perceived workload and increase the risk of occupational stress-related illness [5].

From a public health standpoint, occupational stress in retail workers is not merely an organizational concern; it represents a significant and preventable contributor to population-level mental health burden, work disability, and associated healthcare costs [6]. Empirical evidence from essential retail and grocery workers further confirms that stress accumulation in service occupations translates directly into measurable declines in mental health and workforce sustainability [7]. Psychological workload—the subjective experience of mental, physical, and temporal demands—serves as an early indicator of stress accumulation and is a precursor to burnout, depression, and musculoskeletal disorders if left unaddressed [8,9].

Occupational health psychology frameworks emphasize that workplace stress emerges from dynamic interactions between job demands and available psychological resources [10,11]. The Job Demands-Resources (JD-R) model posits that stress results when psychological demands outweigh resources including physical capacity, social support, and autonomy [12]. Conservation of Resources (COR) theory extends this, proposing that stress occurs when individuals perceive threatened or actual resource loss [13,14]. Both frameworks converge in supporting a multidomain understanding of occupational stress that integrates psychosocial, environmental, and physical capacity factors simultaneously.

Despite the theoretical rationale for multidomain assessment, prior occupational health research has tended to examine isolated stressor domains—focusing either on psychosocial factors [15], physical demands [4], or environmental conditions [5]—without addressing their combined effects on perceived workload. This fragmented approach limits the capacity for early, targeted public health intervention.

Functional physical capacity, assessed via absolute handgrip strength, offers a practically important and field-deployable indicator of workers’ available resources. Grip strength is associated with musculoskeletal health, overall vitality, and vulnerability to both physical and psychological demands [16,17]. Grip strength has increasingly been proposed as a vital sign of health, reflecting overall physical reserve and population-level mortality risk [18]. Crucially, absolute handgrip strength can be obtained rapidly in workplace settings using a standard dynamometer, making it suitable for integration into occupational health screening programs without specialist infrastructure [19]. From a public health screening perspective, grip strength provides an objective, low-cost indicator that can complement self-reported psychosocial data to identify workers at elevated risk before clinical-level problems emerge [20].

Advances in digital health technology offer new opportunities for scalable occupational health surveillance. Progressive Web Applications (PWAs) provide cross-platform compatibility, require no installation, and enable secure, real-time data collection in naturalistic workplace settings [21]. These features are well-suited to ecological momentary assessment principles, capturing workers’ psychological experiences in context [22]. Scoping reviews of mobile applications for workplace health promotion confirm the growing potential of app-based tools for occupational screening, while also identifying usability and evidence quality as key gaps requiring further investigation [23]. Emerging evidence for digital workplace health promotion programs further supports the feasibility of technology-enabled occupational health surveillance at scale [24]. Despite this growing interest, evidence for the validity and usability of PWA-based multidomain assessment platforms in retail populations remains limited.

This study addressed the following primary research question: Which psychosocial, environmental, and functional capacity factors are most strongly associated with perceived psychological workload in retail workers, and can a mobile digital tool reliably assess these factors in the field? The study aimed to: (1) identify multidomain determinants of psychological workload; (2) demonstrate the validity and usability of the Find My Stress PWA as an occupational health screening tool; and (3) provide evidence-based targets for public health intervention in retail workplaces.

## 2. Materials and Methods

### 2.1. Study Design and Participants

A cross-sectional study was conducted among full-time retail service employees working in large-scale home improvement stores across multiple branches in Thailand. Of the 253 participants who completed the full assessment, 124 were male (median age: 33 years, IQR: 27–41) and 97 were female (median age: 31 years, IQR: 24–38) [total *n* = 221 in regression sample; see Section 2.5]. Participants were eligible if they were aged 18–59 years—reflecting the working-age range used in Thai occupational health research and consistent with age- and sex-specific normative grip strength standards for the Thai population [25]—employed for at least three months to ensure task familiarity and stable stressor exposure patterns, and engaged in customer service duties and direct client interaction or merchandise-handling tasks [4]. Employees with acute illness at the time of assessment were excluded to prevent acute health status from confounding subjective workload and grip strength measurements, as transient illness independently elevates perceived effort and reduces grip force. A total of 253 workers completed the full workload and functional capacity assessment. An additional 359 workers participated in the PWA usability evaluation only—a separate, shorter instrument administered to a distinct participant cohort to avoid assessment burden and response fatigue—giving a combined sample of 634 participants across assessment components.

This study was conducted in accordance with the Declaration of Helsinki and approved by the Human Research Ethics Committee of Rangsit University (COA.No. RSUERB2024-109; approved 1 July 2024). All participants provided written informed consent prior to participation.

### 2.2. The Find My Stress Progressive Web Application

The Find My Stress PWA is a field-deployable digital occupational health screening tool developed to enable rapid, multidomain assessment in workplace settings without requiring software installation. The application operates via standard web browsers on smartphones and tablets (iOS 13+; Android 8+; major desktop browsers), and was designed following ergonomic interface principles including high-contrast visual elements, large touch targets (minimum 44 × 44 pixels), and clear navigation pathways.

Key technical features of the PWA include: (a) responsive design optimized for screen sizes from 4.7 to 10 inches; (b) offline functionality enabling data collection in areas with limited connectivity; (c) secure cloud-based database with encrypted data transmission (TLS 1.3 protocol); (d) average assessment completion time of 6–8 min; (e) automatic data validation and quality checks; and (f) real-time synchronization when network connectivity is restored.

The PWA assessment interface is organized into task-specific modules. Workers first select their primary task type—Task 1: Item lookup and retrieval for customers; Task 2: Product recommendation and direct customer service; Task 3: Product restocking and shelf arrangement—before completing relevant assessment items. Variable labels in the data (e.g., Posture1, Bully3, Others3) reflect this task-specific structure, where the appended numeral denotes the task context (1, 2, or 3) in which the rating was provided. For example, ‘Posture1’ refers to perceived postural difficulty during Task 1 (item lookup), while ‘Bully3’ refers to bullying experiences during Task 3 (product restocking). This task-linked structure allows assessment of how stressor profiles differ across job roles, providing more ecologically valid data than generic global ratings. Workers rate each item on standardized scales (5-point or 11-point, depending on the domain), and the application automatically calculates summary scores and flags potential concerns. Handgrip strength values measured prior to PWA completion are entered directly into the application alongside anthropometric data, enabling integrated functional capacity documentation within the same assessment workflow.

Following validation within Rangsit University, the Find My Stress PWA has been adopted by the university’s health services for institution-wide occupational stress monitoring, demonstrating scalability and practical applicability beyond the research context.

### 2.3. Procedure

Data collection was performed on-site during scheduled work shifts. After receiving a standardized brief orientation from trained research assistants, grip strength measurements were first conducted by trained assessors using a calibrated digital dynamometer. Workers then accessed the PWA using personal smartphones or store-provided tablets and completed the assessment in approximately 6–8 min. All responses were encrypted and transmitted to a secure cloud database.

### 2.4. Measures

Subjective Workload Index (SWI). Perceived psychological workload was assessed using the SWI, a validated two-step screening instrument [26,27]. In Step 1, workers rated eight work-related dimensions on an 11-point scale (0–10): six negatively valenced dimensions (fatigue, risks, concentration demands, complexity, work rhythm, and responsibility) and two positively valenced dimensions (interest and autonomy). The SWI composite was computed as (Σ negative items − Σ positive items) ÷ 8, with higher scores reflecting greater perceived workload. Workers scoring ≥ 2.0 proceeded to Step 2, comprising 14 stressor variables rated on a 5-point scale (0–5) across three task-specific contexts (Task 1: item lookup; Task 2: product recommendation; Task 3: product restocking), spanning four domains: (1) Task Duration, (2) Biomechanical (movement constraints, postural difficulty), (3) Physical Environment (thermal discomfort, air quality, dust, noise, vibration, lighting), and (4) Psychosocial (organisational climate, general health, nutrition, workplace bullying). Workers rated six negatively work-related factors, and two motivative work-related factors on an 11-point scale (0–10), with higher scores indicating greater perceived workload for the negative factors, and vice versa for the positive factors. The SWI has demonstrated acceptable internal consistency and validity across multiple occupational settings [26,27]. Its two-step structure improves field assessment efficiency by directing detailed stressor evaluation only to workers with elevated overall workload perceptions.

Psychosocial factors. Task-specific psychosocial demands were assessed including: perceived postural difficulty (Posture1–3), workplace bullying (Bully1–3), organizational climate (Organization1–3), interpersonal demands from others (Others1–3), and general health perception (GeneralHealth1–3). All subscales demonstrated acceptable internal consistency (Cronbach’s α > 0.80).

Environmental discomfort. Environmental conditions were assessed via 5-point ratings of perceived heat, air quality, dust exposure, noise, lighting, and vibration during each task type. Subjective environmental appraisal was selected consistent with transactional stress theory, which emphasizes the psychological significance of individuals’ perceptions of their environment [28].

Musculoskeletal symptoms. Musculoskeletal discomfort experienced during the past month was assessed across body regions including neck, shoulders and upper back, lower back, arms and fingers, wrists and hands, and legs and feet, using a 5-point severity scale consistent with standardized ergonomic assessment protocols [4].

Functional capacity: Handgrip Strength. Bilateral handgrip strength was measured on-site using a calibrated digital dynamometer following a standardized protocol [19]. Grip strength is an established indicator of physical reserve, musculoskeletal health, and work ability [16,17,18]. Participants completed 2–3 maximal-effort trials per hand; the highest value was recorded. Absolute grip strength (kg) was used in all regression analyses as the primary functional capacity variable. For descriptive sex-comparison purposes, grip strength was additionally normalized by body mass index (BMI; kg/m^2^) to derive the HG/BMI index (Table 1) [20], interpreted using age- and sex-specific normative standards for the Thai population [25].

Usability evaluation. Usability of the Find My Stress PWA was assessed in 359 retail employees using a structured questionnaire evaluating clarity, ease of navigation, response accuracy, and perceived usefulness on a 5-point Likert scale.

### 2.5. Statistical Analysis

Data were analyzed using IBM SPSS Statistics Version 26 (IBM Corp., Armonk, NY, USA), a widely validated software platform for health and social science research [29,30]. Descriptive statistics summarized demographic characteristics, SWI scores, environmental and psychosocial factors, musculoskeletal symptoms, and grip strength. Mann–Whitney U tests with effect size r were used to compare sex differences in continuous variables, given that the SWI and psychosocial/environmental variables are measured on ordinal scales and demonstrated non-normal distributions confirmed by Shapiro–Wilk tests [29,31]. Effect size r was calculated as r = Z/√N, consistent with published guidelines for non-parametric effect size reporting [32]. Spearman rank-order correlations examined associations between SWI, environmental, psychosocial, symptom, and functional capacity variables. Hierarchical multiple regression analysis examined predictors of SWI using the Enter method, whereby all variables within each block were simultaneously entered. Block 1 entered grip strength (left hand), age, and gender as covariates to control for functional capacity and demographic characteristics. Block 2 simultaneously added postural difficulty, workplace bullying, thermal discomfort, task duration, and air quality as predictors. Grip strength was additionally retained in Block 2 as a theoretically grounded functional capacity indicator relevant to occupational health screening; VIFs confirmed the absence of multicollinearity across all models (range: 1.03–1.62). Sex-stratified hierarchical analyses were conducted separately for male and female subgroups, with grip strength and age as Block 1 covariates. Because the hierarchical procedure included task-specific variables applicable only to workers performing those tasks, listwise deletion reduced the regression sample to *n* = 221 complete cases (male *n* = 124, female *n* = 97). To assess whether excluded participants (*n* = 32) differed systematically from the retained sample, Mann–Whitney U tests were conducted comparing the two groups across key demographic and outcome variables: age, handgrip strength (left), SWI, postural difficulty, workplace bullying, heat, task duration, and air quality. No statistically significant differences were observed on any variable (all *p* ≥ 0.072), supporting the characterisation of missing data as Missing at Random (MAR) and indicating that listwise deletion is unlikely to have introduced systematic bias into the regression results. Descriptive and correlation analyses were conducted on the full sample (*N* = 253) unless otherwise noted. Statistical significance was set at *p* < 0.05. Effect sizes were reported using Spearman r coefficients and standardized regression coefficients (β). Given the cross-sectional design, regression ‘predictors’ refer to variables statistically associated with SWI and do not imply causal direction.

## 3. Results

### 3.1. Demographic and Physical Characteristics

The sample comprised 253 retail workers. Median age was 33 years (IQR: 27–41) for men and 31 years (IQR: 24–38) for women. Significant sex differences were observed in height, weight, BMI, and grip strength measures, both in absolute and normalized values (all *p* < 0.001), with men demonstrating higher absolute and BMI-normalized grip values than women (Table 1). HG/BMI differed significantly between sexes (left: *p* < 0.001, r = −0.42; right: *p* < 0.001, r = −0.39), confirming the utility of BMI normalization for cross-sex comparisons in this sample.

### 3.2. Perceived Work Strain

Both male and female retail workers reported broadly similar levels of perceived work strain across all SWI subscales, with no statistically significant sex differences observed (all *p* > 0.05; Table 2). Overall SWI scores indicated moderate psychological workload in both groups. Responsibility received the highest ratings and autonomy the lowest, consistent with JD-R theory predictions of high-demand, low-control occupational profiles [12]. Full subscale values are presented in Table 2.

### 3.3. Psychosocial and Environmental Factors by Task Type

Workers reported discomfort across multiple stressor domains—including movement constraints, postural difficulty, thermal conditions, dust exposure, organizational climate, general health, air quality, and noise—with profiles differing meaningfully by task type and sex (Table 3). Postural difficulty was consistently elevated and showed significant sex differences across all three task contexts (Posture1–3; all *p* < 0.05), with women reporting greater postural strain than men, particularly during product restocking (Posture3: *p* = 0.002, r = 0.19). Workplace bullying was reported at higher levels by women during both item lookup (Bully1: *p* = 0.049) and restocking (Bully3: *p* = 0.004, r = 0.18), suggesting that female retail workers may face disproportionate interpersonal stressor exposure across job roles. Thermal discomfort also showed significant sex differences during restocking tasks (Heat3: *p* = 0.049), consistent with evidence that women exhibit lower thermal tolerance thresholds in moderately warm environments. Organizational climate and general health perceptions were rated significantly more negatively by women during both Task 1 and Tasks 2–3, respectively. Overall, these findings indicate that psychosocial and environmental stressor burdens are not uniformly distributed across the retail workforce, and that sex-specific exposure profiles warrant targeted intervention strategies.

### 3.4. Musculoskeletal Complaints

Musculoskeletal complaints were prevalent, with over 73% of workers reporting discomfort in at least one body region (Table 4). Lower-extremity and axial-region symptoms predominated, consistent with the physical demands of prolonged standing, repetitive load handling, and sustained awkward postures inherent in retail work [4]. The relatively lower prevalence of neck and distal upper-limb complaints likely reflects the predominantly gross motor nature of retail tasks. Full regional prevalence data are presented in Table 4.

### 3.5. Correlation Analyses

**Figure 1:**
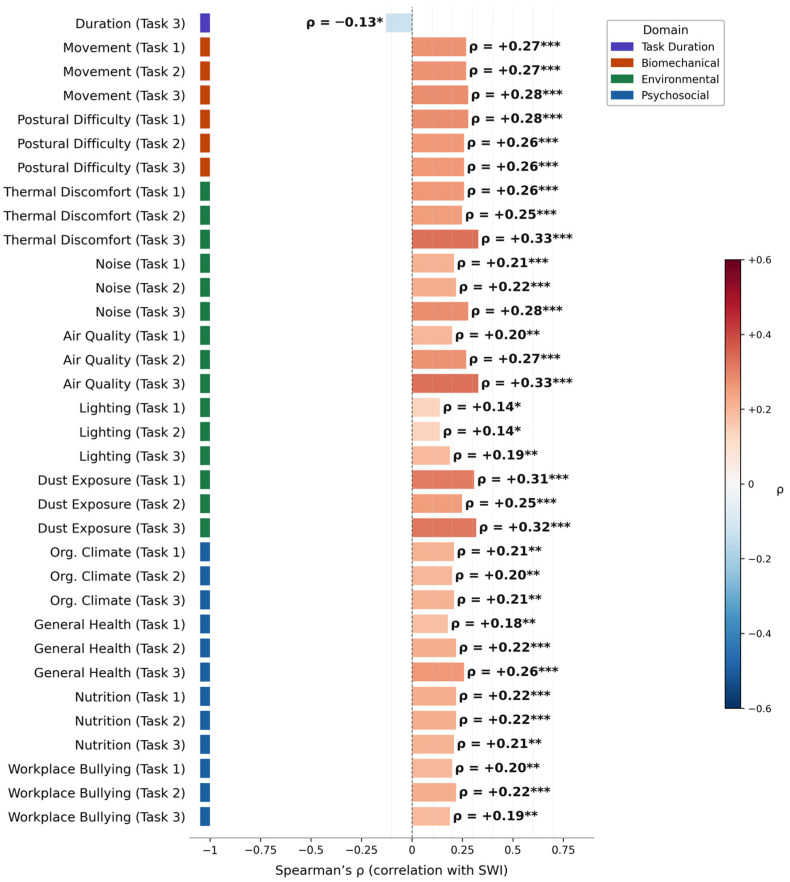
Spearman rank-order correlations between the Subjective Workload Index (SWI) and multidomain occupational stressor variables among retail workers (*N* = 253). Variables are grouped by domain: Task Duration (purple), Biomechanical (orange), Environmental (green), and Psychosocial (blue). Colour intensity reflects correlation magnitude. Task 1 = item lookup and retrieval; Task 2 = product recommendation and customer service; Task 3 = product restocking. * *p* < 0.05; ** *p* < 0.01; *** *p* < 0.001.

Spearman correlations revealed significant associations between SWI and variables across all assessed stressor domains (Figure 1). Among task-specific variables, task duration during restocking (Duration3) showed a significant negative correlation with SWI (ρ = −0.11, *p* < 0.05), while Duration1 (item lookup) was not significantly associated with SWI (ρ = 0.01, *p* = 0.823) and was therefore excluded from the correlation figure. Environmental stressors showed moderate-to-strong positive associations with SWI: air quality and heat during restocking tasks (ρ = 0.33, *p* < 0.001 each) and dust during item lookup (ρ = 0.31, *p* < 0.001) were the strongest environmental correlates. Among psychosocial variables, Nutrition during product recommendation tasks (Nutrition2; ρ = 0.24, *p* < 0.001) emerged as a significant correlate of SWI, alongside interpersonal demands (ρ = 0.30, *p* < 0.001) and general health perceptions (ρ = 0.26, *p* < 0.001). In contrast, grip strength (left hand, absolute value) showed no significant bivariate correlation with SWI (ρ = −0.004, *p* = 0.971), indicating that at the bivariate level, functional capacity was not independently associated with perceived workload. Notably, however, grip strength emerged as a significant independent predictor in the hierarchical multiple regression model, suggesting that grip strength may function as a functional capacity indicator whose contribution to workload is apparent only in the context of a multidomain model, consistent with occupational health screening frameworks [19,20]. Note that Spearman ρ values reflect bivariate SWI associations and are distinct from the Mann–Whitney effect size r values reported in Section 3.1 for sex-group comparisons.

### 3.6. Hierarchical Multiple Regression: Predictors of Psychological Workload

Hierarchical multiple regression analysis was conducted for the full sample and separately for male and female subgroups (Table 5). Block 1 covariates (grip strength, age, and gender) were not significantly associated with SWI in any model (all *p* > 0.79), indicating that functional capacity and demographic characteristics alone did not account for variance in perceived workload. The addition of psychosocial and environmental predictors in Block 2 produced significant incremental variance across all three models (all ΔR^2^ > 0.22, all *p* < 0.001), demonstrating that stressor exposure explained meaningful additional variance beyond the covariates. Variance inflation factors ranged from 1.03 to 1.62, confirming the absence of multicollinearity. Full model statistics are presented in Table 5.

In the full sample Block 2 model, four variables were significantly associated with SWI: postural difficulty (Posture1), workplace bullying (Bully2), task duration (Duration3; negative association), and air quality (Air Quality3; Table 5). Thermal discomfort (Heat3) did not reach significance but was retained per the Enter method protocol. Sex-stratified analyses revealed distinct profiles: postural difficulty and task duration were the only significant associates of SWI among male workers, whereas workplace bullying was the sole significant associate among female workers, suggesting that biomechanical and psychosocial stressor domains have differential relevance by sex. Full coefficients and significance values are reported in Table 5.

Grip strength (left hand) was retained in Block 2 as a functional capacity indicator; VIF values confirmed the absence of multicollinearity across all models (range: 1.03–1.53; Table 5). Although grip strength was not significantly associated with SWI in any model, the positive direction of its coefficient in the full and male models is theoretically noteworthy. Consistent with Conservation of Resources theory [13], workers with greater functional capacity may be systematically assigned to higher-demand tasks—including heavy lifting and extended restocking duties—resulting in elevated perceived psychological workload despite their physical advantage. This pattern suggests that grip strength may serve as a proxy indicator of task demand allocation in retail settings, and its inclusion contributed to overall model stability as evidenced by the low VIF values. From a public health screening perspective, absolute grip strength remains a practical, field-deployable indicator that can be rapidly obtained in workplace settings using a calibrated dynamometer, supporting its integration into routine occupational health assessments [19,20]. Future longitudinal studies should examine whether grip strength predicts incident stress-related illness over time and whether it moderates the relationship between physical stressors and psychological workload in retail and comparable occupational groups.

### 3.7. PWA Usability

Among the 359 retail employees who completed the PWA usability evaluation, 87% provided positive ratings overall. Positive ratings were distributed across all four usability dimensions: clarity of instructions, ease of navigation, accuracy of response options, and perceived usefulness of the tool for reflecting actual work experiences. The Find My Stress PWA demonstrated excellent internal consistency across all assessment items (Cronbach’s α = 0.97), indicating that the composite instrument reliably captured a coherent construct of perceived occupational stress across its multidomain modules. The high usability ratings observed across a workforce with heterogeneous levels of digital literacy—including older workers and those with limited prior experience of web-based health tools—suggest that the PWA’s interface design successfully achieved accessibility without sacrificing assessment depth. These findings support the suitability of the Find My Stress PWA as a scalable, low-burden platform for occupational health surveillance in retail and comparable service-sector environments.

## 4. Discussion

This study identified psychosocial, environmental, and functional capacity determinants of psychological workload among Thai retail workers using a field-deployable digital assessment tool. The findings have direct implications for occupational public health, workplace health promotion, and digital health surveillance.

The hierarchical regression analysis identified postural difficulty as the variable most strongly associated with perceived workload in the full sample model (β = 0.176, *p* = 0.012), and particularly among male workers (β = 0.279, *p* = 0.003). This finding is consistent with ergonomic evidence linking poor biomechanical conditions to heightened strain perceptions [4] and aligns with the JD-R framework [12], which positions physical demands as significant job demands that may co-occur with psychosocial stressors to elevate perceived workload. It should be noted that the cross-sectional design of this study precludes causal inference; the observed association does not establish that postural difficulty causes elevated SWI. From an occupational health standpoint, ergonomic redesign—including workstation adjustment, task rotation, and posture training—represents a plausible and modifiable intervention target for reducing perceived workload burden in retail settings, pending confirmation in longitudinal studies.

Workplace bullying was the second most strongly associated variable with perceived psychological workload in the full sample (β = 0.175, *p* = 0.008), and emerged as the sole significant predictor among female workers (β = 0.283, *p* = 0.006). This pattern is consistent with meta-analytic evidence linking exposure to workplace bullying with psychological distress and musculoskeletal complaints in service occupations [15,33]. The association was particularly evident during product recommendation tasks (Task 2), suggesting that the interpersonal intensity of customer-facing advisory roles may heighten sensitivity to hostile social dynamics. Although the cross-sectional design prevents causal conclusions, these associations underscore the importance of organizational interventions targeting bullying prevention, psychosocial safety climate, and management support as potential public health priorities in high-contact service settings.

Environmental stressors—particularly air quality (β = 0.171, *p* = 0.011) and thermal discomfort (Heat3; β = 0.121, *p* = 0.094)—were associated with higher perceived psychological workload in the full sample model, though thermal discomfort did not reach conventional significance. Retail environments, which feature large semi-open layouts, variable ventilation, and direct weather exposure, may be especially susceptible to environmental stressor accumulation. These associations are consistent with environmental psychology evidence linking thermal and air quality discomfort to cognitive load, irritability, and stress appraisal [5,34], though the cross-sectional design prevents determination of directionality. Prospective studies incorporating objective environmental monitoring—such as WBGT sensors, particulate matter measurement, and sound level meters—are needed to confirm these associations and establish dose–response relationships. Practically, engineering controls such as improved ventilation, heat management, and air filtration represent plausible intervention targets for reducing perceived workload in retail settings.

Grip strength (left hand) was retained in Block 2 on theoretical and practical grounds as a functional capacity indicator, with VIF = 1.53 confirming the absence of multicollinearity. Although grip strength was not significantly associated with SWI in any model (β = 0.039, *p* = 0.605 in the full sample), its positive coefficient direction in the full and male models is theoretically noteworthy. Consistent with Conservation of Resources theory [13], workers with greater physical capacity may be systematically allocated to higher-demand tasks—including heavy lifting and extended restocking duties—which may in turn be associated with elevated perceived workload despite their physical advantage. It should be emphasised that this interpretation is speculative given the cross-sectional design; no causal direction can be inferred from the present data. Notably, grip strength showed no significant bivariate association with SWI (ρ = −0.004, *p* = 0.971), and its non-significance across all hierarchical models suggests it does not function as an independent predictor of psychological workload in this sample. Nevertheless, from a public health screening perspective, absolute grip strength remains a practical, field-deployable functional capacity indicator obtainable with a calibrated dynamometer, and its inclusion in multidomain occupational health assessments may help identify workers with reduced physical reserve who warrant closer monitoring [19,20]. Future longitudinal studies should examine whether grip strength prospectively predicts stress-related illness and whether it moderates associations between physical stressors and psychological workload in retail settings.

The hierarchical multiple regression model accounted for approximately 20% of variance in perceived psychological workload in the full sample (Adjusted R^2^ = 0.199), with Block 2 explaining a significant increment of 22.7% beyond the covariates (ΔR^2^ = 0.227, *p* < 0.001). Sex-stratified models explained 22.4% and 17.0% of variance in male and female subgroups respectively, suggesting that the identified stressor profiles have differential relevance by sex. This level of explained variance is consistent with, and in many cases exceeds, published cross-sectional models of perceived occupational stress, which typically account for 15–30% of variance [10,35]. The remaining unexplained variance reflects the inherently multi-determined nature of psychological workload and indicates that additional contributors—including individual coping styles, personality traits, organisational support quality, and cumulative stressor history—are not captured in a single cross-sectional assessment. These represent important targets for future longitudinal research. The observation that predictors spanning three distinct stressor domains were associated with SWI underscores the value of integrated, multidomain occupational health assessment approaches over siloed single-domain evaluations [35].

The Find My Stress PWA demonstrated excellent internal consistency (α = 0.97) and high usability (87% positive ratings), supporting its validity as an occupational health screening instrument and its acceptability for use in real-world retail environments. The task-specific assessment structure—linking stressor ratings to defined job tasks (item lookup, product recommendation, restocking)—provides a level of ecological validity not achievable with generic global rating instruments, enabling more targeted identification of high-risk work contexts. The PWA’s adoption by Rangsit University’s health services for ongoing occupational stress monitoring further demonstrates translational utility and scalability beyond the research setting.

### Strengths and Limitations

This study presents several strengths. The multidomain assessment approach integrates psychosocial, environmental, and functional capacity data rarely combined in single studies. The inclusion of objective handgrip strength measurement adds a biologically grounded indicator to complement self-report data. The large combined sample (*N* = 634 across assessment components) and the ecological validity of field-based PWA assessment support the generalizability of findings within the Thai retail sector.

Several limitations warrant consideration. First, and most importantly, the cross-sectional design of this study precludes any causal inference; all reported associations reflect statistical relationships observed at a single time point and should not be interpreted as evidence that stressor exposures cause elevated psychological workload. Longitudinal designs are required to establish temporal sequences and directionality. Second, sampling was restricted to large-format home improvement retail stores in Thailand, which may limit generalizability to other retail formats, cultural contexts, or occupational settings [36]. Third, most measures relied on self-report, introducing susceptibility to common method bias and social desirability effects; future research should incorporate objective physiological indicators such as cortisol, heart rate variability, or actigraphy to complement subjective workload ratings. Fourth, environmental conditions were assessed via subjective perception rather than objective instrumentation; while perceptual appraisal is theoretically appropriate per transactional stress theory [28], sensor-based monitoring (e.g., WBGT, sound level meters, particulate matter sensors) would provide stronger evidence for environmental associations and enable dose–response analyses. Fifth, the study did not assess individual difference variables—including personality traits, psychological resilience, coping styles, or work experience—that may moderate stressor-workload associations and account for residual unexplained variance. Sixth, the regression sample was reduced to *n* = 221 due to listwise deletion of task-specific missing data; future studies should employ multiple imputation or mixed-effects models to retain the full sample and improve statistical power. Seventh, the role of grip strength in the hierarchical model warrants replication in independent samples, as its non-significant but theoretically consistent positive coefficient requires prospective investigation before any practical conclusions are drawn. Collectively, these limitations indicate that the present findings should be interpreted as preliminary and hypothesis-generating. Confirmatory longitudinal research in diverse retail formats and cultural contexts is warranted before the identified stressor-workload associations can be generalised or used as a basis for causal intervention recommendations.

## 5. Conclusions

Retail service workers experience complex, interacting psychological stressors across psychosocial, environmental, and functional capacity domains. This study demonstrates that postural difficulty, workplace bullying, thermal discomfort, poor air quality, and reduced functional capacity all independently contribute to perceived psychological workload. These findings reinforce the need for integrated, multidomain occupational health approaches grounded in JD-R and COR theoretical frameworks, with direct implications for workplace health promotion policy and practice.

The Find My Stress PWA provides a validated, scalable, and low-burden digital platform for occupational stress screening and early identification of at-risk workers. Absolute handgrip strength represents a practical, field-deployable functional capacity indicator that can be rapidly integrated into routine occupational health screening without specialist infrastructure, and its inclusion as a functional capacity indicator in the multidomain regression model underscores the value of including objective physical measures alongside self-reported psychosocial data. Together, these approaches support proactive, evidence-based public health interventions targeting psychosocial climate, environmental quality, and worker physical capacity in retail and other high-demand service environments.

Several directions for future research emerge from this study. Longitudinal designs are needed to establish causal pathways between multidomain stressors and psychological workload outcomes, and to determine whether early screening with tools such as the Find My Stress PWA can predict burnout, musculoskeletal disorders, or work disability over time. Intervention studies evaluating the effectiveness of ergonomic redesign, bullying prevention programs, and environmental engineering controls—guided by the stressor profiles identified here—would translate these findings into actionable occupational public health practice. Future studies should incorporate objective physiological measures (e.g., salivary cortisol, heart rate variability, actigraphy) alongside sensor-based environmental monitoring to overcome the common method bias inherent in fully self-reported designs. Cross-cultural replication in retail sectors across Southeast Asia and beyond would test the generalizability of the multidomain stressor model and the PWA platform. Finally, examining the role of grip strength across different occupational groups and task demands will clarify whether this functional capacity indicator has broader utility as a screening variable in integrated occupational health surveillance systems.

## Figures and Tables

**Table 1:** Sex differences in demographic and physical characteristics of retail workers (*N* = 253).

Variable	Men, Median (IQR)	Women, Median (IQR)	U	Z	*p*	r
Age (years)	33 (27–41)	30 (23–37)	13,985	−0.47	0.639	0.03
Height (cm)	170.0 (165–175)	160.0 (156–165)	2344	−8.17	<0.001	0.51
Weight (kg)	73.0 (61.8–85.0)	59.0 (51.9–72.0)	4931	−5.08	<0.001	0.32
Grip Strength Left (kg)	35.2 (26.9–39.8)	26.0 (22.4–28.6)	2495	−9.37	<0.001	0.59
Grip Strength Right (kg)	36.5 (30.6–41.5)	27.7 (25.3–30.9)	2420	−9.51	<0.001	0.60
LHG/BMI	1.46 (1.20–1.68)	1.05 (0.86–1.32)	3747	−5.30	<0.001	0.33
RHG/BMI	1.44 (1.26–1.69)	1.12 (0.96–1.36)	3876	−5.04	<0.001	0.32

Note. Mann–Whitney U test. LHG/BMI = left handgrip/BMI; RHG/BMI = right handgrip/BMI. Effect size r reported.

**Table 2:** Sex differences in workload factors of retail workers (*N* = 253).

Variable	Men, Median (IQR)	Women, Median (IQR)	U	Z	*p*	r
Fatigue	7.0 (6.0–9.0)	8.0 (6.0–9.0)	7335	−1.03	0.303	0.06
Risks	6.0 (5.0–8.0)	6.0 (5.0–8.0)	7658	−0.46	0.645	0.03
Concentration	7.0 (5.0–8.0)	7.0 (5.0–8.0)	7751	−0.30	0.764	0.02
Complexity	7.0 (5.0–8.0)	7.0 (5.0–8.0)	7550	−0.65	0.513	0.04
Work Rhythm	7.0 (5.0–8.0)	7.0 (5.0–8.0)	7718	−0.36	0.720	0.02
Responsibility	8.0 (7.0–9.0)	8.0 (7.0–9.3)	7691	−0.41	0.683	0.03
Interest	7.0 (5.0–8.0)	6.0 (4.0–8.0)	7207	−1.25	0.213	0.08
Autonomy	5.0 (3.0–8.0)	5.0 (3.0–7.0)	7224	−1.22	0.224	0.08
SWI	3.5 (2.9–4.5)	3.6 (2.9–4.6)	7499	−0.73	0.464	0.05

Note. Mann–Whitney U test. SWI = Subjective Workload Index.

**Table 3:** Sex differences in biomechanical, environmental, health, and psychosocial workload-related factors by task type (*N* = 253).

Variable	Task	Men (Mdn)	Women (Mdn)	U	Z	*p*	r
Movement1	1	3.0 (3.0–4.0)	3.0 (3.0–4.0)	5860	−2.20	0.026	0.14
Posture1	1	3.0 (2.0–4.0)	3.0 (3.0–4.0)	5925	−2.10	0.036	0.13
Posture2	2	3.0 (2.0–4.0)	3.0 (2.0–4.0)	5454	−2.57	0.01	0.16
Posture3	3	3.0 (2.0–4.0)	3.0 (3.0–4.0)	4951	−3.03	0.002	0.19
Heat3	3	3.0 (2.0–4.0)	3.5 (3.0–4.0)	5459	−1.97	0.049	0.12
Dust1	1	3.0 (2.0–4.0)	4.0 (2.0–4.0)	5970	−2.00	0.045	0.13
Organization1	1	3.0 (2.0–3.0)	3.0 (2.0–4.0)	5687	−2.56	0.011	0.16
General health2	2	2.5 (1.0–3.0)	3.0 (2.0–4.0)	5704	−2.06	0.04	0.13
General health3	3	2.0 (1.0–3.0)	3.0 (2.0–4.0)	5284	−2.32	0.02	0.15
Nutrition2	2	2.0 (1.0–3.0)	3.0 (2.0–4.0)	5654	−2.15	0.032	0.14
Nutrition3	3	2.0 (2.0–3.0)	3.0 (2.0–4.0)	5331	−2.22	0.026	0.14
Bully1	1	1.0 (1.0–3.0)	2.0 (1.0–3.0)	5981	−1.97	0.049	0.12
Bully3	3	1.0 (1.0–2.0)	2.0 (1.0–3.0)	5007	−2.90	0.004	0.18

Note. Mann–Whitney U test. Task 1 = Item lookup and retrieval; Task 2 = Product recommendation and customer service; Task 3 = Product restocking; Effect size r reported. Only variables with significant sex differences (*p* < 0.05) are presented.

**Table 4:** Musculoskeletal complaints by body region among retail workers (*N* = 253).

Body Region	% Affected
Legs and feet	31.0
Upper back and shoulders	23.4
Lower back and waist	18.9
Arms and fingers	15.8
Wrists and hands	10.5
Neck	6.5

**Table 5:** Hierarchical Multiple Regression Predicting Subjective Workload Index (SWI) Scores (*N* = 221).

		All Workers (*n* = 221)		Male (*n* = 124)		Female (*n* = 97)	
Predictor	Task	β	*p*	β	*p*	β	*p*
***Block 1: Covariates***							
Grip Strength Left	All	0.032	0.698 ns	0.061	0.509 ns	−0.055	0.598 ns
Age	—	−0.014	0.842 ns	0.005	0.961 ns	−0.048	0.646 ns
Gender	—	0.040	0.632 ns	—	—	—	—
*R*^2^; *F*(*df*); *p*		0.001; F(3,217) = 0.094; *p* = 0.963		0.004; F(2,121) = 0.232; *p* = 0.793		0.005; F(2,94) = 0.224; *p* = 0.800	
***Block 2: Predictors†***							
Grip Strength Left	All	0.039	0.605 ns	0.063	0.444 ns	−0.044	0.640 ns
Postural Difficulty	Task 1 ^a^	0.176	0.012 *	0.279	0.003 **	0.050	0.633 ns
Workplace Bullying	Task 2 ^b^	0.175	0.008 **	0.104	0.230 ns	0.283	0.006 **
Thermal Discomfort	Task 3 ^c^	0.121	0.094 ns	0.110	0.238 ns	0.107	0.368 ns
Task Duration	Task 3 ^c^	−0.179	0.004 **	−0.256	0.002 **	−0.089	0.358 ns
Air Quality	Task 3 ^c^	0.171	0.011 *	0.159	0.069 ns	0.182	0.093 ns
Δ*R*^2^; *F*Δ(*df*); *p*		ΔR^2^ = 0.227; F(5,212) = 12.449; *p* < 0.001		ΔR^2^ = 0.264; F(5,116) = 8.378; *p* < 0.001		ΔR^2^ = 0.226; F(5,89) = 5.220; *p* < 0.001	
*VIF range*		1.03–1.57		1.03–1.36		1.03–1.62	
**Adjusted R^2^ (Block 2)**		0.199		0.224		0.170	

Note. Enter method; all predictors simultaneously entered within each block. Block 1: grip strength (left), age, and gender (full model) or grip strength and age (sex-stratified) as covariates. Block 2: grip strength retained as functional capacity indicator (VIF confirmed no multicollinearity); psychosocial and environmental predictors added. † Grip strength included in Block 2 on theoretical grounds as an occupational health screening indicator. Task 1 ^a^ = Item lookup and retrieval; Task 2 ^b^ = Product recommendation and customer service; Task 3 ^c^ = Product restocking. ns = not significant. * *p* < 0.05; ** *p* < 0.01.

## Data Availability

The datasets generated and analyzed during the current study are available from the corresponding author on reasonable request, subject to institutional data sharing policies and ethical approval requirements.
